# The Effects of Modifying the Activity of Nitriding Media by Diluting Ammonia with Nitrogen

**DOI:** 10.3390/ma14092432

**Published:** 2021-05-07

**Authors:** Mihai Ovidiu Cojocaru, Mihai Branzei, Andrei Mihai Ghinea, Leontin Nicolae Druga

**Affiliations:** 1 Department of Metallic Materials Science, Physical Metallurgy, Faculty of Materials Science and Engineering, University POLITEHNICA of Bucharest, 060042 Bucharest, Romania or mocojocaru2005@yahoo.co.uk (M.O.C.); amihai724@yahoo.com (A.M.G.); 2 Section IX-Materials Science and Engineering, Technical Sciences Academy of Romania, 030167 Bucharest, Romania; ld@uttis.ro; 3 Research Department, UTTIS Industries SRL, 077185 Vidra, Romania

**Keywords:** nitriding, ammonia–nitrogen atmosphere, experimental programming, nitrogen potential

## Abstract

This paper discusses the issue of the effects of modifying the activity of nitriding media by diluting ammonia with nitrogen and the concomitant variation in the degree of ammonia dissociation on the layer’s growth kinetics and their phase composition. To understand and quantify the effects of the variation in the main parameters that influence the layer growth kinetics, the experimental programming method was used and mathematical models of interactions between influence and kinetics parameters were obtained for two metallic materials: Fe-ARMCO and 34CrAlMo5 nitralloy steel. It was concluded that the nitriding operating temperature and the degree of nitrogen dilution of the ammonia have statistically significant influences on the kinetics of the nitrided layer. In the same context, it was analytically proved and experimentally confirmed that the ammonia degree dissociation from the gaseous ammonia-nitrogen mixture, along with the dilution degree of the medium with nitrogen, significantly influences the nitrogen potential of the gaseous mixture used for nitriding and thus the concentration of nitrogen in balance at the medium thermochemically processed metal product interface.

## 1. Introduction

The analysis of the equilibrium in the Fe-N system allows obtaining information regarding the phase composition of the nitrided layers obtained under strict conditions of temperature and nitrogen concentration in the furnace chamber atmosphere [1,2,3,4,5]. The adjustment of the nitrided layer phase composition can be set by altering the nitrogen potential of the atmospheres used: by maintaining it at the level of nitrogen solubility in a certain phase, one is able to control the phase composition of the layer [6,7,8,9,10,11]. Thus, a nitrided layer can be obtained, which in the areas adjacent to the surface is composed exclusively of solid solution *α**_N_***, or *γ**’***, or *ε* (with a certain concentration of nitrogen), depending on the requirement (operational destination of the nitrided product). In the case of radiation heating, the phase composition of the layer can be controlled by the dilution of the ammonia with nitrogen (or other inert gases, e.g., argon), by products of the preliminary dissociation of ammonia, oxygen, and atmospheric air, by performing nitriding in vacuum, or at high pressures, or by diluting ammonia with gaseous mixtures containing carbon. In the last case, the nitriding process is transformed into ferritic nitrocarburizing, in the superficial areas of the layer, adjacent to the medium, observing the presence of carbonitrides [12]. The control of the nitriding process and implicitly of the results can be ensured by a multitude of methods; among them, the ammonia atmosphere degree dissociation or dilution occupies a leading place [2,3,13,14,15,16,17,18,19]. The easiest ways to change the nitrogen potential of the medium used in nitriding, in the case of radiation heating, are those by diluting ammonia with nitrogen or with products of prior dissociation of ammonia, such as oxygen, atmospheric air, water vapor. In the case of nitrogen dilution up to 80–90% [6,7,18], a considerable reduction in the fragility of the surface area of nitrides can be ensured. The size of the internal nitrided zone (the diffusion zone) is not affected in this case or may even register a slight increase [6]. Another advantage of dilution with nitrogen (or argon) is the saving of ammonia, while considerably reducing the danger of explosion. Diminishing the fragility of the nitrided layer can be ensured by a denitriding step performed in an atmosphere of fully dissociated ammonia. In this way, the removal of excess nitrogen from the layer is ensured, this being practically the one that did not form nitrides with the alloying elements. By diluting the partially dissociated ammonia with other gases, layers with an extremely diversified phase composition can be obtained. Thus, if in the case of a partially dissociated ammonia atmosphere, at 570 °C, *α*_NH3_ = 30%, layers with a phase composition can be obtained in accordance with the Fe-N thermodynamic equilibrium diagram by using an atmosphere consisting of 30% NH_3_, 70% N_2_ and H_2_ (mixture made by the prior ammonia dissociation), the nitrided zone will be composed exclusively of the *γ**’*** phase, and when increasing the mixture proportion (N_2_-H_2_) obtained by prior dissociation of ammonia up to 80%, the area of nitrides in contact with the medium-layer interface will disappear completely. At the same time, the diffusion zone (internal nitriding) will be composed of *α*_N_ [8,9].

The use of ammonia mixtures with products of its previous dissociation, can ensure for nitriding steels with medium or low carbon content layers without the presence of ε phase, thus ensuring an increase in fatigue strength by 85% [6]. Nitriding in an atmosphere of ammonia diluted with oxygen-containing gases intensifies the diffusion of nitrogen [19]. The composition of the gas mixture is adjusted so that the nitrogen potential of the atmosphere is greater than 3 (*P_NH3_/P_H2_^3/2^ > 3*) and the oxygen or water vapor content of the atmosphere is below the limit at which iron oxidation occurs. The oxide film formation ensures a wear and corrosion resistance increase in nitrided products. Vacuum nitriding assures a much faster growth kinetic nitrided layer compared to atmospheric pressure [20].

In general, research on the effect of pressure variation below and above atmospheric pressure (respectively, vacuum, or high pressure nitriding) indicates an intensification of the growth kinetics of nitrided layers [20,21]. Along with these variants of intensifying the growth kinetics of nitrided layers, the use of laser radiation [22,23] at low pressures [20,21] in the presence of rare earths [24] and thermogas cycling [25] represent other variants of particularly efficient processing.

## 2. Materials and Methods

Experimental research has aimed to highlight the influences of changes in the chemical composition of gaseous media used in nitriding with radiation heating on the phase composition of nitrided layers and their growth kinetics. The aim was to highlight and quantify the singular and cumulative effects of the influence of nitriding temperature, the degree of ammonia dissociation and the degree of nitrogen dilution of the nitriding medium through experimental programming. Achieving these goals has become possible by keeping constant the ratio of the ammonia and nitrogen proportions, respectively, at values imposed by the adopted research program and changing the degree of ammonia dissociation by varying the overall flow of the gas mixture. The programming method used in the research was the non-compositional 2nd order (*K* = 3) [26,27,28].

The materials on which the investigations have been carried out were Fe-ARMCO and 34CrAlMo5 (W1.8507) nitralloy steel. The complete chemical analysis carried out on the 34CrAlMo5 steel bars from which the samples were taken for the experiments was the following: 0.43% C; 0.64% Mn; 0.32% Si; 1.12% Cr; 0.92% Al; 0.33% Mo; 0.2% Ni.

The samples with dimensions of 6 mm × 10 mm × 20 mm of the two materials after degreasing with isopropyl alcohol were nitrided in partially dissociated ammonia (*α***_NH3_** ϵ [45 ÷ 70%]) and diluted with nitrogen (60% max), at different temperatures in the range 540 ÷ 620 °C, for 4 h holding time.

The nitriding was carried out in a vertical electric oven (UTTIS INDUSTRIES SRL, Vidra, Romania) with Φ190 mm × 600 mm retort dimensions, 8 KW installed power, provided with automatic temperature adjust and control system. The samples of 34CrAlMo5 nitralloy steel prior to nitriding were subjected to hardening (870 °C/oil), followed by high tempering (580 °C/air). The ammonia degree dissociation was controlled by the titration method.

To perform optical metallography (OM), the samples were etched with Nital 3% reagent.

The OM analyses were performed on an image analysis system consisting of a Reichert UnivaR microscope (C. REICHERT AG., Wien, Austria), a Polaroid DMC Ie RGB camera and Buehler Omnimet Enterprise Software (version V5.0, BUEHLER, Ltd., Lake Bluff, IL, USA). A PhenomWord ProX microscope (Phenom-World B.V, Eindhoven, The Netherlands) was used for SEM-EDS microscopy, with light optical magnification range 20–134×, electron optical magnification 80–130,000×, maximum acceleration voltages of 15 kV, a backscattered electron detector (fully integrated) and energy dispersive X-ray spectrometer detectors (fully integrated), with a nominal resolution of 10 nm, at room temperature.

The estimation of the total thickness of the nitrided layer (the area of compounds to which the size of the diffusion area is added, i.e., the internal nitriding area) was made differently for the two materials taken in the analysis. Thus, for Fe-ARMCO, the total thickness of the nitrided layer was assessed in cross section, as the distance from the sample surface to the boundary of the area where excess γ ‘(Fe_4_N) phase separations are still observable, and for 34CrAlMo5 nitralloy steel was highlighted by the selective chemical etching of the different areas where nitrogen is present or not; thus, in the case of this nitrided steel, the diffusion zone (internal nitriding zone) is much more intensely etched compared to the areas unaffected by nitrogen diffusion.

To verify the nitrogen content in the marginal areas of the nitrided samples (obtained analytically and by SEM-EDS microscopy), expression of the nitrogen content in the medium under certain processing conditions, the gases resulting from the combustion of Fe-ARMCO foils (thickness of 0.1 mm), were analyzed. A LECO TC-236 type equipment (LECO Corporation, St. Joseph, MO, USA) was used, complying with the requirements of ASTM E1019-18 [29].

The layers obtained by nitriding under different conditions were also subjected to measuring Vickers microhardness using a micro-Vickers hardness tester model CV400 (TECNIMETAL, Madrid, Spain), applying a normal load of 200 gf/300 gf and a dwell time of 10 s. The hardness values were the average of ten indentations. The indentations were performed at 150 microns from the surface of the layer.

## 3. Results and Discussion

The effect quantification of the gaseous nitriding parameters’ variation in a partially dissociated ammonia atmosphere (70% max), diluted with nitrogen (60% max), was performed by experimental programming, respectively, the active experiment method, using a 2nd order non-compositional program (*K* = 3). It should be noted that in experimental research, regardless of the matrix nature (Fe-ARMCO or 34CrAlMo5 nitralloy steel), gaseous nitriding in a partially dissociated ammonia atmosphere additionally diluted with nitrogen was performed under the same conditions, as shown in Table 1, 4 h holding time. Cooling was performed in an ammonia atmosphere up to 250 °C and then in air.

As independent variables (*X_i_*), the process temperature, the ammonia dissociation degree, and the degree of dilution with nitrogen, were chosen, respectively, and as the dependent variable (*Y*) of the process, the total thickness of the nitrided layer was chosen.

The connection between the coded values of the independent variables (*X_i_*) and the decoded-natural ones (*Zi*), respectively, is given by the correlation:

(1) $$X_{i}=(Z_{i}-Z_{i_{0}})/\Delta Z_{i}$$

where: *Xi* represents the coded value of the independent parameter in the analysis;

*Z_i_* is the natural value of the independent parameter in the analysis;

Δ*Z_i_* is the variation interval of the independent parameter in the analysis.

The experimentally obtained data were processed under the conditions imposed by the adopted programming method, as shown in Table 1.

The results of the statistical processing of the coefficients of the regression equations related to the Fe-ARMCO (Table 2) and, respectively, of the concordance of the calculated nonlinear model (Table 4) led to their particular form: Equation (2). After the same route, the data were obtained for 34CrAlMo5 nitralloy steel, these being presented in Tables 4 and 5, respectively, which led to their particular form: Equation (21).

(2) $$Y=\delta_{tot}=767.3+226.5X_{1}-97.4X_{1}X_{3}+92.8X_{1}{}^{2}-85.8X_{3}{}^{2}$$

where: *X_1_* and *X_3_*, respectively, represent the codified forms of the independent parameters: temperature (*X_1_*) and the proportion of nitrogen (*X_3_*) in the gaseous mixture used for nitriding.

*Y* is the actual value of the total thickness of the nitrided layer.

The significance of the statistical parameters contained in Table 2 is as follows:

$S_{0}^{2}$ represents the dispersion of the experiment reproducibility (dispersion of the experimental data), expressed by the equation:

(3) $$S_{0}^{2}=\frac{\sum_{1}^{n}(\Delta Y_{u}{)}^{2}}{\nu_{2}}$$

where: Δ*Y* represents the difference between a certain value of the dependent parameter *Y* and its arithmetic mean value between the ***n*** experiments performed in identical conditions.

*ν_2_ = n* − 1 represents the number of degrees of freedom.

*n* represents the number of experiments performed under identical conditions.

$S_{b_{i}}^{2}$ represents the dispersion in determining the values of the coefficients of the regression equation:

(4) $$S_{b_{i}}^{2}=\frac{S_{0}^{2}}{\sum_{u=1}^{N}X_{i_{u}}^{2}}$$

$\Delta b_{i}$ represents the confidence interval corresponding to each calculated coefficient of the regression equation.

Note that *α* represents the significance threshold (*α* = 0.05), and $S_{b_{i}}$ the quadratic mean deviation with which the coefficient *b**_i_*** is calculated.

(5) $$\Delta b_{i}=t_{\alpha ;N}S_{b_{i}}$$

where: *t* represents the value corresponding to the Student criterion (tabulated value according to *α* and *N*).

*N* represents the number of experiments corresponding to the type of experiment program adopted (N = 15, Table 1).

A coefficient of the regression equation is considered statistically determined if its absolute value is greater than the absolute value of its confidence interval, so if it satisfies the condition:

(6) $$|b_{i}|\;\geq \;|\Delta b_{i}{|}_{\;}$$

The statistical verification results of the concordance of the computed non-linear model according to Equation (2), presented in Table 3, and similarly the statistical concordance of the non-linear model related to the 34CrAlMo5 nitralloy steel, according to Equation (21)-Table 7, confirm that, with a probability of 95%, these express the correlation between the independent parameters considered (the technological maintaining temperature and the proportion of nitrogen in the ammonia–nitrogen mixture) and the total size of the nitrided layer obtained for the two metallic materials.

$F_{c}$ represents the expression of the Fischer criterion and $S_{conc}^{2}$ the dispersion caused by the calculated mathematical model, expressed as follows:

(7) $$F_{c}=\frac{S_{conc}^{2}}{S_{0}^{2}}$$

(8) $$S_{conc}^{2}=\frac{\sum_{u=1}^{N}({\bar{Y}}_{u}-Y_{u_{\exp}}{)}^{2}}{N-k^{’}}$$

where: $\bar{Y_{u}}$ represents the value of the dependent parameter ***Y*** calculated using the regression Equation (2) or (21) under the conditions corresponding to the experiment *u*.

$Y_{u_{\exp}}$ represents the value of the dependent parameter Y actually obtained in the experimental conditions corresponding to the experiment *u.*

*N* represents the total number of experiments.

k’ represents the number of coefficients of the regression equation (including the free term).

**Figure 1:**
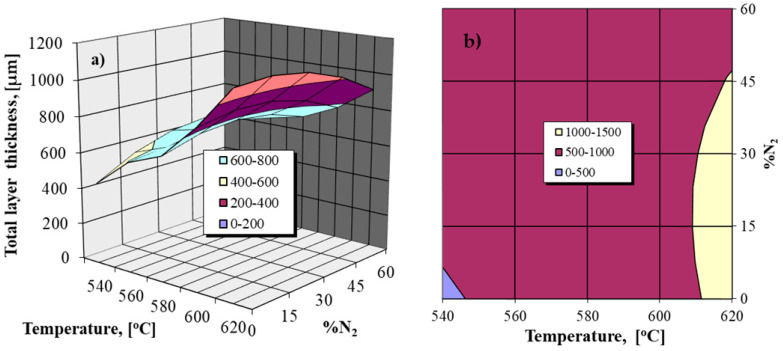
The dependence of the total thickness of the nitrided layer on the nitriding temperature and the nitrogen proportion in the NH_3_ + N_2_ gaseous mixture for Fe-ARMCO; 4 h holding time; (**a**) the response area of the regression Equation (2); (**b**) iso-properties domains.

The analysis of the obtained regression Equation (2) and its graphic expressions shown in Figure 1 highlights the relatively reduced effect of the nitrogen proportion variation in the ammonia–nitrogen gaseous mixture used for nitriding on the total thickness of the nitrided layer. Thus, there are slight increases in the total thickness of the nitrided layer for relatively low degrees of nitrogen dilution (up to 30%), the excess of this dilution value involving a slight decrease in the thickness of the nitrided layer.

Note the strong effect of nitriding temperature variation on the general growth kinetics of the layer. Another observation is related to the fact that the variation in the ammonia dissociation degree (within the limits of 20–70%) in the NH_3_ + N_2_ gaseous mixture does not statistically affect the growth kinetics of the nitrided layer.

For thermodynamic reasons related to the ammonia decomposition reaction, Kogan and Solodkin [9,30] conclude that in the case of nitriding in an atmosphere of ammonia diluted with nitrogen (infrared heating), the nitrogen potential of the atmosphere depends on the ammonia dissociation degree, α, the proportion of ammonia in the initial mixture, ***γ***, and the proportion of the dilution gas, nitrogen (100 *λ*), as shown in Equation (9):

(9) $$\Pi_{N}=\frac{\lambda (1-\alpha )(1+\alpha \lambda )}{{\left(1.5\alpha \lambda \right)}^{3/2}}=\frac{\left(100-\%N_{2})(1-\alpha )[1+\alpha (100-\%N_{2})\right]}{{\left[1.5\alpha (100-\%N_{2})\right]}^{3/2}}$$

Note: The initial gaseous mixture consists exclusively of ammonia and nitrogen (%NH_3_ + %N_2_ = 100%).

**Figure 2:**
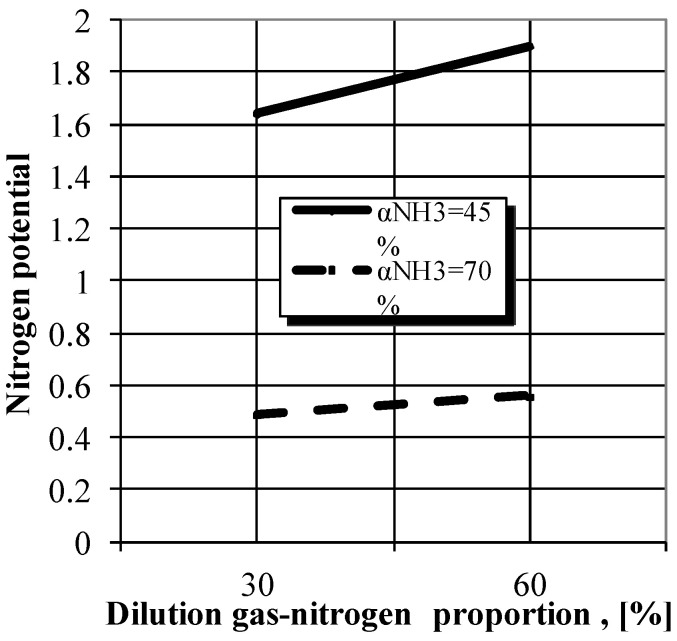
The N potential variation in the NH_3_ + N_2_ gaseous mixture used for nitriding, depending on the NH_3_ dissociation degree (45% and 70%) and the gas-nitrogen dilution proportion.

It follows that noticeable effects regarding the variation in nitrogen potential are recorded when the ammonia dissociation degree in the ammonia-nitrogen gas mixture changes. Thus, for a 30% nitrogen dilution degree of the nitriding medium, the decrease in ammonia dissociation from 70% to 45% leads to an increase in nitrogen potential by about 235% (about 240% for a dilution grade of 60%), as shown in Figure 2, while for the same degree of ammonia dissociation (45% or 70%), increasing the degree of nitrogen dilution of the medium from 30% to 60% induces a significantly smaller change in the nitrogen potential (increase by about 14/15%, so with an order of magnitude below that recorded at the variation in the ammonia dissociation degree).

The immediate implications of this conclusion, confirmed by our own experimental research, are related to the phased composition of the nitrided layer and related to the nitrogen concentration of the ε phase. The *ε*-Fe_2–3_N phase is formed because of the reaction between phase ***γ’*** (Fe_4_N) and phase ***ζ*** (Fe_2_N) [9,30,31], the reaction being:

(10) $${\left[Fe_{4}N\right]}_{\varepsilon }+NH_{3}=2{\left[Fe_{2}N\right]}_{\varepsilon }+3/2H_{2}$$

Note: The *ε* index shows that the both nitrides are parts of the ε-Fe_2–3_N solid solution, based on Fe_2–3_N nitride.

The reaction constant of Equation (10) can be expressed by means of its free energy, Δ*G*^0^, as in Equation (11):

(11) $$\mathrm{lg}K_{1}=\frac{\Delta G^{o}}{4.47T}$$

Or, taking into account the iron nitride forming energy [11], in the simplified form:

(12) $$\mathrm{lg}K_{1}=-\frac{2670}{T}+2.93$$

On the other hand, as Lahtin and Kogan have shown [9,30]:

(13) $$K_{1}=\frac{a_{\varepsilon }}{a_{\gamma^{’}}}\times \frac{1}{\Pi_{N}}$$

where: Π*_N_* represents the atmosphere nitrogen potential;

*a_ε_* and, respectively, *a**_γ’_*** nitrogen activities of the nitride phase *ε* (Fe_4_N and Fe_2_N).

Accepting that the phase *ε* represents an ideal solid solution consisting of Fe_4_N and Fe_2_N nitrides, nitrogen activities in the two nitrides can be replaced by molar fractions [9,30].

As *N^’^_γ_/N_ϛ_ =* 1, the last expression of the equilibrium constant will have the form like in Equation (14):

(14) $$\frac{N_{\zeta }{}^{2}}{1-N_{\zeta }}=\Pi_{N}\times K_{1}$$

Kogan and Solodkin [10,11] started from the expression of the relationship that allows the determination of the concentration of nitrogen in the *ε* phase, an expression that takes into account the nitride masses, *m_γ’_* and *m_ζ_’*, respectively, as well as their molar masses, *M_γ_’* and *M_ζ_*, respectively; the result is a simplified reaction that links the concentration of nitrogen in the *ε* phase, *[N]_ε_*, to the molar participation of Fe_2_N nitride, *N_ζ_*, in the composition of *ε* nitride:

(15) $${\left[N\right]}_{\varepsilon }=\frac{0.06}{1-0.47N_{\zeta }}\times 100\%$$

Replacing Equation (11) in Equation (14) and later Equation (11) in Equation (15), a system of equations will result (16):

(16) $$\left\{ \begin{array}{l} \frac{N_{\zeta }{}^{2}}{1-N_{\zeta }}=\Pi_{N}\times 10^{-\frac{2670}{T}+2.93}\\ {\left[N\right]}_{\varepsilon }=\frac{0.06}{1-0.47N_{\zeta }}\times 100 \end{array}\right.$$

**Figure 3:**
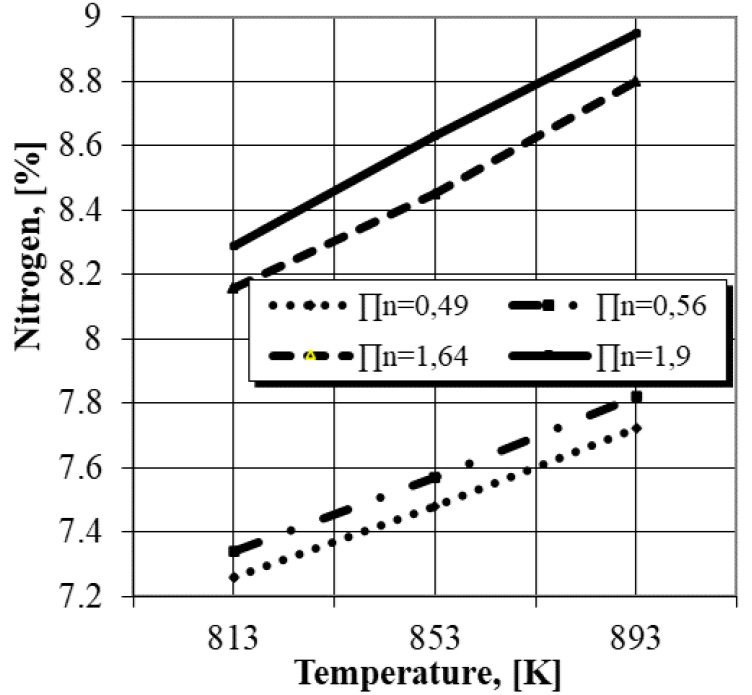
The N_2_ concentration modification in the surface areas of the nitrided layer, depending on the nitriding temperature and the N_2_ potential of the gaseous mixture.

From this system of two equations results the value of the nitrogen concentration in the marginal area of the layer (*ε* phase) corresponding to a certain nitrogen potential of the environment. The results on the nitrogen concentration in the marginal area of the nitrided layer obtained by processing in gaseous mixtures with different nitrogen potentials at different temperatures, determined by following the algorithm developed above, are presented in Figure 3.

From the analysis of the obtained results presented in Figure 3, it is found that in the conditions of relatively low nitrogen potentials (0.49, 0.56), ensured by high degrees of ammonia dissociation (70%) and relatively low proportions of nitrogen (about 30%) in the NH_3_ + N_2_ gaseous mixture, the N_2_ concentration in the surface areas exceeds 7 wt.% (value confirmed by the analysis of the gases resulting from the combustion of Fe-ARMCO foils processed under the same conditions), thus creating the premise for the *ε* phase appearance.

**Figure 4:**
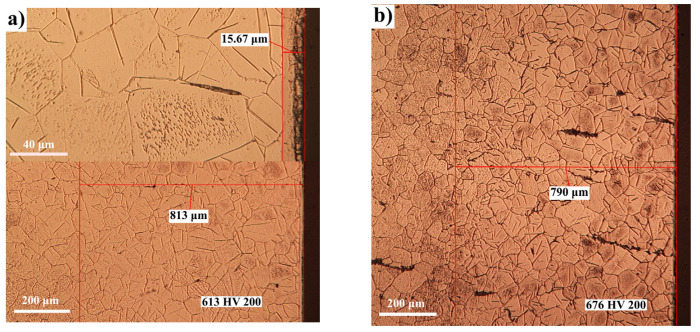
OM of nitrided Fe-ARMCO at 580 °C/4 h in an atmosphere of ammonia diluted with nitrogen in a proportion of 30% (**a**), respectively, 60% (**b**); degree of ammonia dissociation: 45% (**a**) and 70% (**b**); nitrogen potentials: 1.64 (**a**) and 0.56 (**b**); N_2_ concentrations in the surface areas of the layers: 8.5% (**a**) and 7.58% (**b**). The microhardness indentations were performed at 150 microns from the surface of the layer.

**Figure 5:**
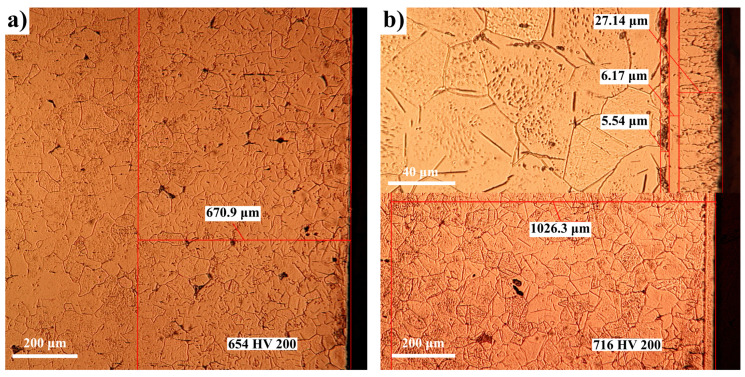
OM of the nitrided Fe-ARMCO at different temperatures: 540 °C (**a**) and 620 °C (**b**) within atmosphere of partially dissociated ammonia, α_NH3_ = 45%, diluted with nitrogen into 60% ratio; N_2_ potential 1.9; ~8.3% N_2_ (**a**) and ~8.9% N_2_ (**b**) at the surface layers. The microhardness indentations were performed at 150 microns from the surface of the layer.

The OM studies presented in Figure 4 and Figure 5 for Fe-ARMCO confirm the previous statements. In Figure 4a, a detail is presented at the top, so that the morphology of the actual layer can be observed; the average thickness of the layer itself is 15.67 microns, compared to the total thickness, which is about 813 microns. The average microhardness measured at about 150 microns from the surface has comparable values: 613 HV for a nitrogen potential of 1.64 and 676 HV for a nitrogen potential of 0.56, respectively.

The effect of the nitriding temperature variation, in the conditions of keeping constant the ammonia dissociation degree and the proportion of the dilution component in the gaseous mixture (N_2_), is felt mainly in the growth kinetics of the nitrided layer and in the phase’s types/number.

The nitriding temperature increase over the eutectoid transformation temperature in the Fe-N system finally implies, after the slow cooling, the braunite appearance (*α_N_ + γ^’^*), as shown in the detail at the top of Figure 5b, it being formed at a maximum depth of 5.54 microns.

Thus, in the Fe-N system, below the eutectoid transformation temperature (590 °C), the microstructural analysis shown in Figure 4 and Figure 5a, respectively, highlights the sequence of phases:

$$\varepsilon +\gamma^{’}\to \gamma^{’}\to \alpha_{N}+\gamma_{excess}^{’}\to \alpha$$

They have different sizes and proportions, depending on the concentration of nitrogen, being dependent on the ammonia dissociation degree and the degree of dilution with nitrogen. At temperatures above 590 °C, as shown in Figure 5b, in the sequence of phases the appearance of eutectoid type braunite is observed, which is consistent with the statement of Lahtin [6]:

$$\varepsilon +\gamma^{’}\to \gamma^{’}\to braunite(\alpha_{N}+\gamma^{’})\to \alpha_{N}+\gamma_{excess}^{’}\to \alpha$$

Concurrently, due to the enhancement of the nitrogen diffusion coefficient in *α* and *ε* phases, there is a substantial increase in the total thickness of the nitrided layer, with rates depending on the particular processing conditions (ammonia dissociation degree; degree of dilution with nitrogen), as shown in Figure 4 and Figure 5.

Concluding, in the layer section, from the surface to the interior, as shown in detail in Figure 5b, the following sequence appears: the first substrate, with a thickness of 27.14 microns, is composed of *ε + γ^’^_excess_*; is followed by the one consisting only of the *γ* phase, with a thickness of 6.17 microns; then follows the substrate composed only of braunite (5.54 microns), so that up to about 1026 microns the presence of compounds with needle morphology can be observed in the microstructure.

By nitriding 34CrAlMo5 nitralloy steel, totally different results are obtained in terms of the layer growth kinetics (Table 1) and their phase composition, compared to Fe-ARMCO. The differences are generated by the presence of alloying elements (Cr, Al, Mo), but also of C cumulated with the effect of their presence on the diffusion coefficient of nitrogen in the alloyed matrix.

Analytical [9], the value of the diffusion coefficient in the alloyed steel matrix can be calculated, following the following algorithm:

The diffusion coefficient value ($D_{N}^{\eta_{i}}$) in the alloyed matrix is calculated, considering the type and proportion of alloying elements and the afferent coefficients ($\eta_{EA}^{D_{N}}$):

(17) $$D_{N}^{\eta_{i}}=\eta_{1}^{D_{N}}.\eta_{2}^{D_{N}}\ldots \ldots \eta_{i}^{D_{N}}$$

where: ***D_N_*** represents the N_2_ diffusion coefficient in Fe-ARMCO;

and

(18) $$\eta_{1\ldots i}^{D_{N}}=\frac{D_{N}^{\eta_{1\ldots i}}}{D^{N}},$$

The coefficients are related to the alloying elements (1, …, *i*), depending on their actual concentration in steel, the value of which can be calculated with Equation (19) or determined in the graphical expressions of dependencies $\ln\eta_{i}^{D_{N}^{\alpha }}=f(\%EA)$ [9], for the case that the nitrogen diffusion occurs in solid solution *α*, or $\mathrm{lg}\eta_{i}^{D_{N}^{\varepsilon }}=f(\%EA)$, the equation given by the same author for the case that nitrogen diffusion takes place in the *ε* phase (compound area of the layer):

(19) $$\ln\eta_{i}^{D_{N}}=\frac{B{\left(\%EA\right)}^{n}}{T}$$

where: *B* and *n* are coefficients.

Calculate the diffusion coefficient of nitrogen in the allied matrix ($D_{N}^{{\left(n_{1\ldots i}\right)}^{\alpha ;\varepsilon }}$):

(20) $$D_{N}^{{\left(n_{1\ldots i}\right)}^{\alpha ;\varepsilon }}=\eta_{1}^{D_{N}^{\alpha ;\varepsilon }}.\eta_{2}^{D_{N}^{\alpha ;\varepsilon }}\ldots \ldots \eta_{i}^{D_{N}^{\alpha ;\varepsilon }}.D_{N}^{\alpha ;\varepsilon }$$

Note: the relation is identical for the two cases, respectively, for calculating the diffusion coefficients in *α* or *ε* phases.

The calculated values of the nitrogen diffusion coefficients in *α* solid solution, respectively, in the *ε* solid solution, for the case of the 34CrAloMo5 nitralloy steel are presented in Table 4 and Table 5.

It should be noted that, under the conditions of an alloy matrix, as is the case of the 34CrAlMo5 nitralloy steel matrix, the nitrogen diffusion in the *ε* or *α* phase is much slowed by the presence of alloying elements, mostly with high affinity for nitrogen. It was found that the value of the nitrogen diffusion coefficient in the *α* solid solution of a non-alloy matrix (e.g., in Fe-ARMCO) is over 43 times higher, as shown in Table 4, compared to that corresponding to the diffusion in the alloy matrix of steel, in the temperature range 540–620 °C, and almost eight times higher in the case of diffusion in the *ε* solid solution, according to Table 5.

All these calculated differences are confirmed by the results of experimental research presented in Table 1 and OM measurements performed on the 34CrAlMo5 nitralloy steel and, on the Fe-ARMCO, respectively.

The particular form of the regression Equation (21), statistically confirmed (see Table 6), representing the mathematical model of the interaction between the processing parameters taken into analysis, respectively, the nitriding temperature, the degree of ammonia dissociation and the nitrogen ratio in the ammonia/nitrogen mixture, on the one hand, and the size of the total nitrided layer, on the other hand, is similar to that obtained in the case of nitrided Fe-ARMCO, according to Equation (2), confirming that the variation of the ammonia degree dissociation in the range of 20–70% does not affect statistically significant nitrided layer growth kinetics in the context of nitrogen dilution of the atmosphere.

(21) $$Y=\delta_{tot}=177.5+47.02X_{1}-14.47X_{3}+47.75X_{1}{}^{2}$$

**Figure 6:**
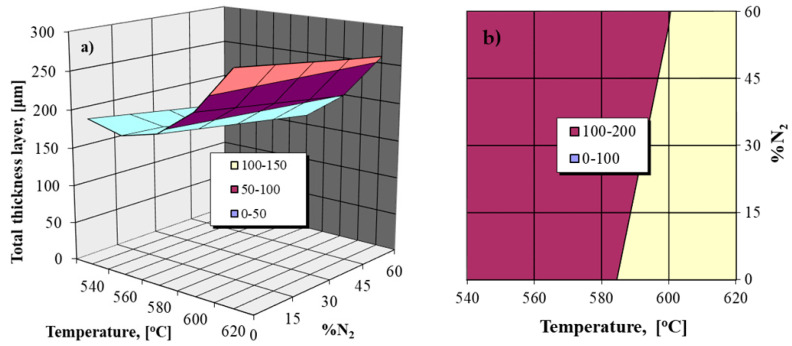
The dependence of the total thickness of the nitrided layer on the nitriding temperature and the nitrogen proportion in the NH_3_+N_2_ gaseous mixture for the 34CrAlMo5 nitralloy steel; 4 h holding time; (**a**) the response area of the regression Equation (21); (**b**) iso-properties domains.

In the absence of dilution gas (nitrogen, argon, etc.), experimental research shows a significant influence of the variation of this quantity on the layer growth kinetics [6,7,8,18,32]. The mathematical model obtained, according to Equation (21), passed the concordance test as it is presented in Table 7, and its graphical expressions, drawn in Figure 6, express quite suggestively the effect of the variation of the two parameters with statistically significant influence, temperature, and ammonia dilution degree on the total thickness of the nitrided layer.

With the help of the regression Equations (2) and (21), by calculation, the total thickness of the nitrided layer can be anticipated in the conditions in which the parameters for carrying out the thermochemical processing are specified. Assuming, for example, that the processing of 34CrAlMo5 nitralloy steel takes place at a temperature of 560 °C/4 h holding time in the atmosphere of ammonia diluted with 45% N_2_, it is required to anticipate by the calculation of the total thickness of the nitrided layer. In this sense, it is necessary to encode the values of the parameters *X_1_* and *X_3_*, (Equation (1) was used), and by entering the values encoded in Equation (22) and solving it, the thickness (*Y*) of 158.7 microns will be obtained.

In conclusion, the sequence of steps to be completed in order to obtain the second-order regression-polynomial Equation (22) (mathematical models of the interactions between the parameters of interest concerned—in this case, the total thickness of the nitrided layer), after establishing the concrete experimentation program (Table 1) according to the type of programming adopted, would be:

(22) $$Y=b_{0}+\sum_{i=1}^{k}b_{i}x_{i}+\sum_{\begin{array}{l} i=1\\ j=1\\ j\neq k \end{array}}^{k}b_{ij}x_{i}x_{j}+\sum_{i=1}^{k}b_{ii}x_{i}^{2}$$

which represents the general form of the equation.

Determination by the calculation of the regression equation coefficients, $b_{0}$, $b_{i}$, $b_{i}{}_{j}$, $b_{i}{}_{i}$, taking into account the actual results obtained.

So:

(23) $$b_{0}=1/n_{0}\sum_{n_{0}}^{n_{0}}y_{0}{}_{u}$$

The free term coefficient is determined from several a number of $n_{0}$ experiments in identical conditions, on the basic level of the independent variables, and representing the values of the dependent parameters in these identical experimental conditions.

(24) $$b_{i}=A\sum_{y=1}^{N}x_{i_{u}}y_{u}$$

The coefficients of the term $x_{i}$; $x_{i_{u}}$ represent the coded value of the independent parameter $x_{i_{u}}$ in experiment *u*, and $y_{u}$ represents the natural value of the dependent parameter *y* in experiment *u**.***

(25) $$b_{i}{}_{j}=D\sum_{u=1}^{N}x_{i_{u}}x_{j_{u}}y_{u}$$

The coefficients of the term $x_{ij}$

(1) The dispersion determination of the experiment reproducibility denoted with $S_{0}^{2}$(see Equation (3)) is conducted by performing three experiments at the basic levels of the independent parameters, $x_{1}=x_{2}=x_{3}=0$.
(2) The dispersion calculation in determining the coefficients of regression equations $S_{b_{0}}^{2}$, $S_{b_{i}}^{2}$, $S_{b_{ii}}^{2}$, $S_{b_{ij}}^{2}$.

So:

(26) $$S_{b_{0}}^{2}=\frac{S_{0}^{2}}{n_{0}}$$

(27) $$S_{b_{i}}^{2}=AS_{0}{}^{2}$$

*A* = 1/8 for *k* = 3—number of independent variables.

(28) $$S_{b_{ii}}^{2}=\left(B+\frac{1}{p^{2}.n_{0}}\right)S_{0}{}^{2}$$

*B* = 1/4; *p* = 2; *n*_0_ = 3 for *k* = 3.

(29) $$S_{b_{ij}}^{2}=D.S_{0}{}^{2}$$

*D* = 1/4 for *k* = 3.

(1) Statistical verification of the coefficients of the nonlinear model is performed by comparing the absolute values of the calculated coefficients of the regression equations, with the values corresponding to their confidence intervals (calculated with Equation (5)); only those coefficients that meet the condition of statistical verification will remain in the particular forms of Equation (6).
(2) The verification of the concordance hypothesis of the adopted nonlinear model is performed with the Fischer criterion (*F*), comparing its calculated value (*F_calc_*) with the tabulated value (*F_tab_*). The calculated value of the Fischer criterion, Equation (7), contains the dispersion produced by the regression equation, $S_{conc}^{2}$, Equation (8); it is considered that the programming method is chosen correctly, so the determined quadratic equation reflects with maximum probability (95%, for an α = 0.05) the connection between the independent variables taken in the analysis (*X*, …, *X_i_*) and the dependent ones (*y*), only if the condition *F_calc_ < F_tab_*.

It is also of interest to find out the proportion of nitrogen introduced into the atmosphere used for nitriding, so that in conditions of 580 °C/4 h holding time, resulting in a total layer thickness of 185 microns (for the same steel), in Equation (21) the real value of the independent parameter *Y* (185) and the coded one of the independent variable *X_1_* will be introduced, and will result in the coded value of the variable *X_3_*, which by subsequent decoding, will lead to the value of about 14.5% N_2_.

**Figure 7:**
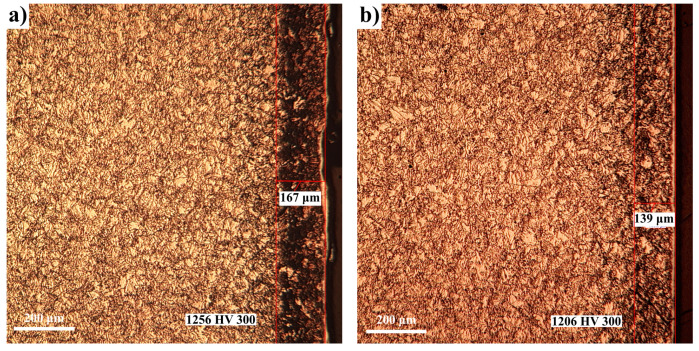
OM images of the 34CrAlMo5 nitralloy steel to 580 °C for 4 h holding in atmosphere of ammonia diluted with 30% N_2_ (**a**), respectively, 60% N_2_ (**b**); the ammonia dissociation degree: 45% (**a**) and 70% (**b**); potential of nitrogen: 1.64 (**a**) and 0.56 (**b**); nitrogen concentration in the superficial layer: 8.5% (**a**) and 7.58% (**b**). The microhardness indentations were performed at 150 microns from the surface of the layer.

**Figure 8:**
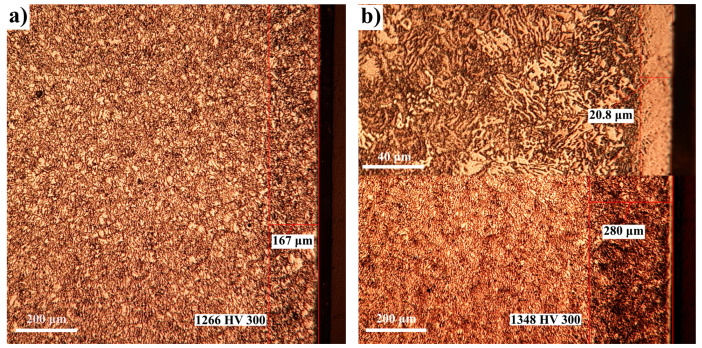
OM images of 34CrAlMo5 nitralloy steel nitrided to various temperatures: 540 °C (**a**) and 620 °C (**b**) in atmosphere of partially dissociated ammonia, *α*_NH 3_ = 45%, diluted with 60% N_2_; potential of nitrogen 1.9; nitrogen concentrations in superficial areas of layers: 8.3% (**a**) and 8.9% (**b**). The microhardness indentations were performed at 150 microns from the surface of the layer.

The results of the calculation regarding the change in the diffusion coefficient value in the *α* phase of the 34CrAlMo5 nitralloy matrix steel, according to Table 4, justify the values of 4 to 5 times lower the total layer thickness, presented in Figure 6, Figure 7 and Figure 8, obtained in the same conditions of Fe-ARMCO processing.

On the other hand, the thickness of the compound area related to 34CrAlMo5 nitralloy steel is also smaller compared to that obtained in the case of Fe-ARMCO, the differences also being determined by a substantial reduction in the diffusion coefficient in the *ε* phase, according to the data presented in Table 5. The phase composition of the nitrided layer obtained in the case of 34CrAlMo5 nitralloy steel, under different thermochemical processing conditions, will be in terms of phase sequence like that obtained in the case of Fe-ARMCO, the differences being imposed by the presence of nitrides of alloying elements and their carbides along with iron nitrides. Thus, in the case of 34CrAlMo5 nitralloy steel at temperatures below 590 °C (see Figure 7 and Figure 8a from phase *ε*–[(Fe,M)_2–3_N]), it separates on cooling, *γ’* in excess [(Fe,M)_4_N] phase, and attached to it, towards the deeper areas of the layer, we will find *ε + γ’ + α*. Note: M represents the alloying elements.

The concurrent presence of the *ε* and *α* phases is attributed to the displacement of the *ε* phase on the grain boundaries. The internal diffusion zone, the majority part of the layer and which is etched intensely, consists of the solid solution of nitrogen in ferrite, deficient in alloying elements, M_3_C type carbides and *γ’* in excess [(Fe,M)_4_N] phase. At temperatures above the eutectoid transformation temperature (Figure 8b), in the nitrided layer the mechanical mixture eutectoid, respectively, braunite, i.e., *α_N_* + *γ’*-(Fe,M)_4_N also appears.

The alloying element nitride formation ensures a substantial increase in the 34CrAlMo5 nitralloy steel’s surface layer microhardness, at values up to 1256 HV 300 in the conditions of nitriding at 580 °C/4 h/*α*_NH3_ = 45% ÷ 30%N_2_, compared to about 600 HV 200 in the case of Fe-ARMCO under the same conditions.

The OM images performed on the nitrided 34CrAlMo5 nitralloy steel samples are presented in Figure 7 and Figure 8, certifying the presence of the nitrided layer, whose phase composition is in full agreement with the conditions in which the thermochemical processing took place.

In Figure 7a, because of the selective chemical etching (Nital 3%), the total thickness of the formed layer was highlighted (167 microns) at a nitrogen potential of 1.64. In Figure 7b, at a nitrogen potential of 0.56, the layer formed has about 139 microns thickness, thus registering a decrease. The microhardness average values are comparable: 1256 HV compared with 1206 HV.

In Figure 8b, a detail is presented at the top, so that the morphology of the actual layer can be observed; the average thickness of the layer itself is 20.80 microns, compared to the total thickness, which is about 280 microns. It is observed that an increase in the temperature, from 540 °C to 620 °C, leads simultaneously both to an increase in the total layer thickness, from 167 to 280 microns, which means over 65%, and to an increase in the microhardness values, from 1266 HV to 1348 HV, which means over 6%.

## 4. Conclusions

Mathematical models of the interactions between thermochemical processing parameters, nitriding temperature, ammonia dissociation degree, nitrogen ratio in the nitriding atmosphere and the total thickness of the nitrided layer, for the two metallic materials analyzed, Fe-ARMCO and 34CrAlMo5 nitralloy steel, respectively, allow the prediction by the calculation of the total thickness of the nitrided layer under certain strictly specified conditions, or if a certain value is imposed on this quantity, the determination of how to choose variables of statistical significance, respectively, nitriding temperature and dilution degree with nitrogen of the atmosphere so that it can be obtained.

As novelty elements, experimental research aimed to highlight the effects of the concurrent variation in the nitrogen dilution degree of the atmosphere used for nitriding and the ammonia dissociation degree on the growth kinetics of the layer. Thus, analytically it was concluded that these two parameters significantly influence the nitrogen potential of the gas mixture used for nitriding and, implicitly, the nitrogen concentration in equilibrium at the interface between the environment and the thermochemically processed metal product, but experimentally it has been shown that, in the case of an ammonia–nitrogen gaseous mixture, the variation in ammonia dissociation degree in the gaseous mixture does not statistically significantly influence the growth kinetics of the nitrided layer, and the kinetic can be modified by the degree of dilution.

The presence of alloying elements’ effect on the nitrogen diffusion coefficient in the *α* and *ε* phase, respectively, was calculated and it was concluded that its value significantly decreases in the presence of alloying elements, which justifies the much smaller size of nitrided layers obtained on 34CrAlMo5 nitralloy steel compared to Fe-ARMCO, nitrided under the same conditions.

## Figures and Tables

**Table 1:** The 2nd order non-compositional program (*K* = 3); correspondence between natural and codified values of the independent parameters; actual conditions for carrying out the experiences and the results.

Factors	Temperature Process, [°C]		Dissociation Degree of NH_3_, [%]		Properties of N_2_ in Gas Mixture, [%]		Total Layer Thickness Y, [μm]	
	Natural Units, [°C], Z_1_	Encode Values, X_1_	Natural Units, [°C], Z_2_	Encode Values, X_2_	Natural Units, [°C], Z_3_	Encode Values, X_3_	Fe-ARMCO	34CrAlMo5
Base level	Z_o_ = 580	0	Z_o_ = 45	0	Z_o_ = 30	0	-	-
Variation range	ΔZ = 40	-	ΔZ = 25	-	ΔZ = 30	-	-	-
Top level	Z_o_ + ΔZ = 620	+1	Z_o_ + ΔZ = 70	+1	Z_o_ + ΔZ = 60	+1	-	-
Lower level	Z_o_ – ΔZ = 540	−1	Z_o_ – ΔZ = 20	−1	Z_o_ – ΔZ = 0	−1	-	-
Experiment 1	620	+1	70	+1	30	0	991.9	253.3
Experiment 2	620	+1	20	−1	30	0	954.7	238.04
Experiment 3	540	−1	70	+1	30	0	561.4	191.12
Experiment 4	540	−1	20	−1	30	0	573.6	170.76
Experiment 5	620	+1	45	0	60	+1	1026.3	280.1
Experiment 6	620	+1	45	0	0	−1	1224	313
Experiment 7	540	−1	45	0	60	+1	670.9	166.34
Experiment 8	540	−1	45	0	0	−1	479	180
Experiment 9	580	0	70	+1	60	+1	790.2	138.86
Experiment 10	580	0	70	+1	0	−1	682	172
Experiment 11	580	0	20	−1	60	+1	799.9	176.93
Experiment 12	580	0	20	−1	0	−1	696	213
Experiment 13	580	0	45	0	30	0	768.9	175.53
Experiment 14	580	0	45	0	30	0	720	190
Experiment 15	580	0	45	0	30	0	813	167

**Table 2:** Results of statistical processing of experimental data on nitriding Fe-ARMCO in partially dissociated ammonia atmosphere, diluted with nitrogen.

No	Statistical Parameter	No	Statistical Parameter
1	S_o_^2^ = 2164.2	6	t _0.05;15_ = 2.131
2	S_bo_^2^ = 721.4	7	$\left|\Delta b_{1}\right|=\left|\Delta b_{2}\right|=\left|\Delta b_{3}\right|=\pm 110.8$
3	S_b1_^2^ = S_b2_^2^ = Sb_3_^2^ = 270.5	8	$\left|\Delta b_{11}\right|=\left|\Delta b_{22}\right|=\left|\Delta b_{33}\right|=\pm 57.1$
4	S_b12_^2^ = Sb_13_^2^ = Sb_23_^2^ = 541	9	$\left|\Delta b_{12}\right|=\left|\Delta b_{13}\right|=\left|\Delta b_{23}\right|=\pm 49.5$
5	S_b11_^2^ = S_b22_^2^ = Sb_33_^2^ = 721.4	10	$\left|\Delta b_{0^{’}}\right|=\pm 57.2$

**Table 3:** Concordance verifying between the calculated non-linear model Equation (2) and the experimental results according to Table 1.

Y = f(X_1_; X_2_; X_3_)	S_conc_^2^	F_calc_	F_tab_
Equation (1)	9810	4.53	19.39

**Table 4:** The calculation results of the nitrogen diffusion coefficient in the 34CrAlMo5 nitralloy steel *α* solid solution, at different temperatures.

*T*, [°C]	$D_{N}^{\alpha }$ [cm^2^/s]	$\ln\eta_{EA}^{D_{N}^{\alpha }}$					$\Pi \eta_{EA}^{D_{N}^{\alpha }}$	$D_{N_{EA}}^{\alpha }$ [cm^2^/s]	$\frac{D_{N}^{\alpha }}{D_{N_{EA}}^{\alpha }}$
		$\ln\eta_{Al}^{D_{N}^{\alpha }}$	$\ln\eta_{Mo}^{D_{N}^{\alpha }}$	$\ln\eta_{Cr}^{D_{N}^{\alpha }}$	$\ln\eta_{Ni}^{D_{N}^{\alpha }}$	$\ln\eta_{Mn}^{D_{N}^{\alpha }}$			
540	6.2 × 10^−8^	1.62	−0.55	−0.6	−0.75	−0.25	2.3×10^−2^	1.428 × 10^−9^	43.42
580	1.07 × 10^−7^							2.46 × 10^−9^	43.49
620	1.77 × 10^−7^							4.07 × 10^−9^	43.49

**Table 5:** The calculation results of the nitrogen diffusion coefficient in the 34CrAlMo5 nitralloy steel *ε* solid solution, at different temperatures.

*T*, [^o^C]	$D_{N}^{\varepsilon }$ [cm^2^/s]	$\mathrm{lg}\eta_{EA}^{D_{N}^{\varepsilon }}$					$\Pi \eta_{EA}^{D_{N}^{\varepsilon }}$	$D_{N_{EA}}^{\varepsilon }\times \;10{-}^{11}$ [cm^2^/s]	$\frac{D_{N}^{\varepsilon }}{D_{N_{EA}}^{\varepsilon }}$
		$\mathrm{lg}\eta_{Al}^{D_{N}^{\varepsilon }}$	$\mathrm{lg}\eta_{Mo}^{D_{N}^{\varepsilon }}$	$\mathrm{lg}\eta_{Cr}^{D_{N}^{\varepsilon }}$	$\mathrm{lg}\eta_{C}^{D_{N}^{\varepsilon }}$	$\mathrm{lg}\eta_{Mn}^{D_{N}^{\varepsilon }}$			
540	8.55 × 10^−11^	0.5	−0.11	−0.2	−1.1	0	0.12589	1.07	7.99
580	2.39 × 10^−10^							3.00	7.96
620	6.08 × 10^−10^							7.65	7.94

**Table 6:** Results of the statistical processing of the experimental data referring to 34CrAlMo5 nitralloy steel in nitrogen diluted ammonia atmosphere.

No	Statistic Parameter	No	Statistic Parameter
1	So^2^ = 135.16	6	t_0.05;15_ = 2.131
2	S_bo_^2^ = 45	7	$\left|\Delta b_{1}\right|=\left|\Delta b_{2}\right|=\left|\Delta b_{3}\right|=\pm 8.75$
3	S_b1_^2^ = S_b2_^2^ = Sb_3_ = 16.89	8	$\left|\Delta b_{11}\right|=\left|\Delta b_{22}\right|=\left|\Delta b_{33}\right|=\pm 14.29$
4	S_b12_^2^ = Sb_13_^2^ = Sb_23_ = 33.79	9	$\left|\Delta b_{12}\right|=\left|\Delta b_{13}\right|=\left|\Delta b_{23}\right|=\pm 12.38$
5	S_b11_^2^ = S_b22_^2^ = Sb_33_^2^ = 45	10	$\left|\Delta b_{0^{’}}\right|=\pm 14.29$

**Table 7:** Verification of the compliance between the calculated non-linear pattern according to Equation (21) and experimental results according to Table 1.

Y = f(X_1_; X_2_; X_3_)	S_conc_^2^	F_calc_	F_tab_
Equation (21)	454.2	3.36	19.40

## Data Availability

The study did not report any data.
